# Intravascular Lymphoma with Progressive CNS Hemorrhage and Multiple Dissections

**DOI:** 10.1155/2020/6134830

**Published:** 2020-01-10

**Authors:** Ricky Chen, Gurjeet Singh, J. Scott McNally, Cheryl A. Palmer, Adam de Havenon

**Affiliations:** ^1^Providence Brain & Spine Institute, Providence St. Vincent Medical Center, 9135 SW Barnes Road, Suite 461, Portland, OR 97225, USA; ^2^Legacy Medical Group Neurology, Salmon Creek Medical Center, 2121 NE 139th St., Suite 205 Medical Office Building A, Vancouver, WA 98686, USA; ^3^Department of Radiology and Imaging Sciences, University of Utah, 30 N 1900 E, Salt Lake City, UT 84132, USA; ^4^Department of Pathology, University of Utah, Huntsman Cancer Institute, 2000 Circle of Hope Dr., Salt Lake City, UT 84112, USA; ^5^Department of Neurology, University of Utah, 175 N Medical Dr., Salt Lake City, UT 84132, USA

## Abstract

**Introduction:**

Intravascular lymphoma (IVL) is an uncommon and often fatal disease characterized by intraluminal proliferation of lymphomatous cells within blood vessels. Because of a heterogeneous clinical presentation and lack of sensitive diagnostic protocols, diagnosis of IVL is most often made at autopsy. However, with early diagnosis and appropriate chemotherapy, the prognosis is greatly improved and complete remission is possible. In order to broaden the possible presentations of IVL, we present a patient with an atypical manifestation of biopsy-proven intravascular large B-cell lymphoma who suffered dissections of both intracranial and extracranial arteries in addition to progressive intracranial hemorrhages. *Case Report*. A 47-year-old woman presented with unilateral paresthesias. She developed progressive multifocal infarcts and hemorrhage with dissections of both intracranial and extracranial arteries, resulting in coma. Brain biopsy revealed IVL. She received aggressive chemotherapy and remains in complete remission with good neurologic recovery.

**Conclusion:**

IVL is known to exert its pathology on small arteries and capillaries, but is not known to cause dissections of large vessels. The diagnosis should be considered in cases with unexplained arterial dissections and progressive strokes. Early diagnosis with appropriate laboratory screening and tissue confirmation by biopsy can lead to greatly improved outcomes.

## 1. Introduction

Intravascular lymphoma (IVL) is recognized by the World Health Organization (WHO) as an extremely rare subtype of diffuse extranodal large B-cell non-Hodgkin's lymphoma with an estimated annual incidence of 0.5 cases per 1,000,000 [1]. IVL leads to intraluminal proliferation of neoplastic lymphoid cells in small and medium vessels [2]. These cells have lost the ability for transvascular migration due to defects in adhesion molecules [3]. IVL can involve any organ system, particularly the central nervous system (CNS), and its protean neurological manifestations often hinder diagnosis. Patients can present with subacute encephalopathy or dementia, paralysis, seizures, or multifocal cerebrovascular events, by order of frequency [1, 4, 5]. Systemic symptoms vary, but fever and skin lesions are the most common, whereas lymphadenopathy is usually absent [2].

Serologic abnormalities include anemia (63%), high lactate dehydrogenase (LDH) (82%) and beta 2-microglobulin levels (86%) [6]. Bone marrow involvement occurs in 32% of patients, and cerebrospinal fluid (CSF) rarely contains malignant cells [6]. Magnetic resonance imaging (MRI) is nonspecific and can be normal, though there are numerous reports of rapid progression of cerebral infarcts [5]. Organ biopsy is the gold standard for diagnosis of IVL but is frequently performed too late, and cases with CNS involvement are most often diagnosed post-mortem (60%) [7]. Random skin biopsies may help make the diagnosis earlier, with up to 83% sensitivity even without skin findings on exam [8, 9]. While IVL can have a grim prognosis with average untreated survival only a few months from diagnosis, chemotherapies containing rituxumab and doxorubicin have improved three-year survival rates to 60%, which may be further increased when methotrexate is added [1, 10]. When diagnosed and treated promptly, complete remission is seen in up to 42% of cases, although recent survival data with adequate treatment are lacking [11]. We describe an unusual presentation of IVL with multiple dissections of both intracranial and extracranial blood vessels and progressive intracranial hemorrhages (ICH).

## 2. Case Report

A 47-year-old woman with a history of delayed gastric emptying presented with migratory paresthesias traveling up her right side. These episodes occurred daily, lasted 20 minutes, and were associated with headache and confusion. During the month preceding her admission, she was admitted to an outside hospital with right-sided paresthesias, worsening migraines with visual aura, and somnolence. Initial contrast-enhanced MRI of the brain did not have acute findings, and showed minimal background nonspecific T2-weighted-fluid-attenuated inversion recovery (FLAIR) hyperintensities in the subcortical white matter.

After concern of a focal motor seizure, the patient was transferred to our hospital. She had no lymphadenopathy, skin lesions, or edema. Neurologically, she was initially somnolent but easily arousable, and once alert remained interactive and oriented sufficient to participate in the Montreal cognitive assessment (MOCA). Her MOCA score was 22/30 with notable deficits in attention, language and abstraction – a marked departure from her high-functioning baseline. The remainder of her neurologic exam was unremarkable. Laboratory evaluation including cerebrospinal fluid analysis was unrevealing (Table 1).

**Figure 1:**
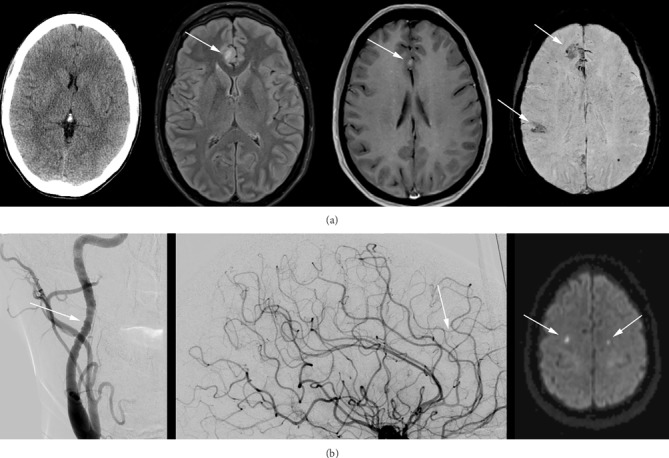
(a) Left to right: computed tomography (CT), fluid-attenuated inversion recovery (FLAIR), T1 post-contrast, and susceptibility-weighted imaging (SWI) 13 days after admission showed minimal findings on CT, but FLAIR hyperintensities and petechial hemorrhages on T1 and SWI images (arrows). These had progressed compared to initial imaging (not shown). (b) Left to right: Carotid and cerebral angiography showing irregular corrugated and beaded appearance of the cervical carotid arteries and subtle intracranial vessel narrowing (arrows), and two new foci of restricted diffusivity on diffusion weighted imaging (DWI) in the bilateral centrum semiovale consistent with interval infarcts (arrows). Note: there was another infarct in the left centrum semiovale on an adjacent slice not shown above.

**Figure 2:**
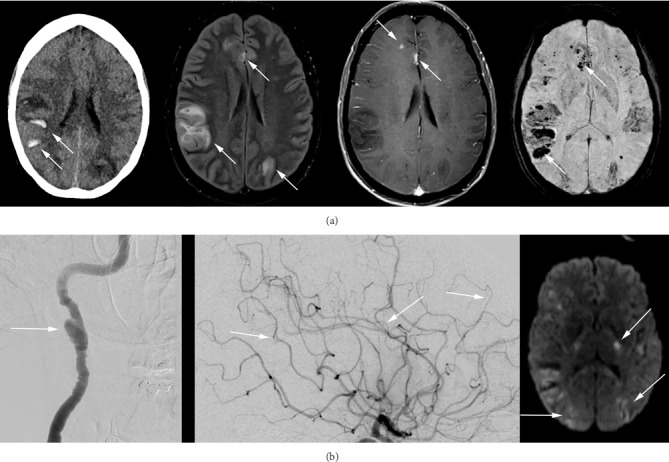
(a) Left to right: Repeat CT, FLAIR, T1post, and SWI showing marked interval progression of hemorrhages on CT and SWI with surrounding edema on FLAIR and subtle foci of enhancement on T1 post-contrast images (arrows). The vasogenic edema and moderate-sized right temporoparietal hemorrhage caused cerebral edema and midline shift, and there were multiple additional petechial hemorrhages. There was also new sulcal FLAIR signal with subtle enhancement consistent with blood-brain barrier breakdown. (b) Left to right: Repeat catheter angiography showed a new right cervical internal carotid artery dissection on carotid angiography (arrow), progression of luminal irregularities in the distal the middle cerebral artery and the anterior cerebral artery branches (arrows), and new areas of cytotoxic edema/infarction on DWI (arrows).

MRI of the brain was repeated 13 days after the initial scan (Figure 1(a)), showing two new foci of restricted diffusivity in the left centrum semiovale consistent with interval infarcts. There was also progression of FLAIR hyperintensities in the right anterior cingulate gyrus and inferior right and posterior left frontal lobes, with corresponding areas of petechial hemorrhage on T2^∗^-weighted imaging. Continuous electroencephalograms (EEG) confirmed encephalopathic slowing without epileptiform discharges. Cerebral angiography showed irregular corrugated and beaded appearance of the cervical carotid arteries, thought to be consistent with chronic fibromuscular dysplasia (Figure 1(b)). High-resolution vessel wall MRI did not show arterial wall enhancement to suggest vasculitis. 18F-fluorodeoxyglucose positron emission tomography (FDG-PET) scan of the brain (Figure 2) and computed tomography (CT) of the chest, abdomen, and pelvis were unrevealing.

**Figure 3:**
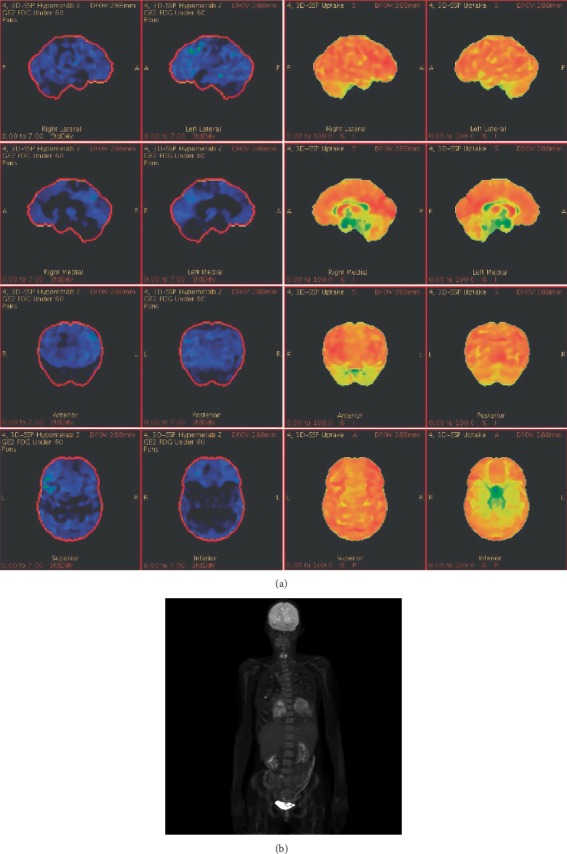
(a) FDG-PET scan of the brain demonstrated mildly diminished FDG uptake in the left more than right bilateral temporal, frontal, and parietal lobes as well as the in the posterior cingulate, precuneus and the primary visual and visual association cortices. The most prominent metabolic reduction was in the left frontal association cortex. There were no focal areas of hypermetabolism in the mesial temporal lobes or elsewhere. This diffuse pattern of mild metabolic changes was not specific for any inflammatory disease or other process, and most suggestive of a global encephalopathy with asymmetry due to more pronounced deficits in the left hemisphere. (b) PET scan of the body was notable for bilateral pleural effusions as well as pericardial effusion and scattered nonspecific ill-defined hypermetabolic pulmonary nodules with differential including infectious/inflammatory process or pulmonary lymphoma with max SUV (standard uptake value) of 6.8.

As an extensive workup was thus far negative (CSF OCB, flow, cytology, HSV/VZV, ANA, ANCA, Mayo paraneoplastic panel, VGKC, AQ4, NMDA, tox screen, EEG without obvious seizure), the patient received a presumptive diagnosis of idiopathic encephalitis and was treated empirically with a five-day trial of intravenous methylprednisolone followed by a prednisone taper. She improved clinically for one month after discharge and returned to work, but gradually developed worsening headache, vomiting, lethargy, and episodic left facial droop. Repeat MRI (Figure 3(a)) showed marked interval progression with new areas of cytotoxic edema/infarction, vasogenic edema, and a moderate-sized right temporoparietal hemorrhage causing cerebral edema and midline shift, with additional petechial hemorrhages on T2^∗^-weighted imaging. There was extensive new sulcal FLAIR signal and enhancement compatible with blood-brain barrier breakdown.

CSF analysis demonstrated marked protein elevation (140 mg/dL) without pleocytosis. Repeat catheter angiography showed a new right cervical internal carotid artery dissection in the distal extracranial portion as well as progression of luminal irregularities indicative of progressive vasculopathy (Figure 3(b)). Magnetic resonance angiography (MRA) of the head and neck the same day revealed additional bilateral vertebral artery dissections involving the entire right vertebral artery and the left V3/4 segments, as well as new narrowing of the bilateral temporal arteries. There was no instrumentation above the level of the common carotid arteries or vertebral artery origins during the initial catheter angiogram to explain this finding. Our differential diagnosis expanded to include atypical vasculitides, inflammatory amyloid angiopathy, complicated Posterior reversible encephalopathy syndrome (PRES), and reversible cerebral vasoconstriction syndrome (RCVS), or IVL (Table 2). Because an elevated LDH level in an atypical case without infection can be suggestive of IVL, we measured LDH and found hers to be elevated to 450 U/L (Table 1).

**Figure 4:**
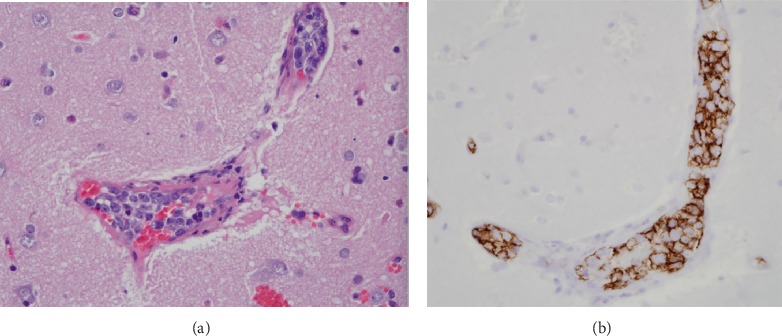
(a) Hematoxylin-and-eosin of the brain biopsy showed small cortical vessels pathologically distended with neoplastic lymphocytes (200X). (b) These cells were positive by immunohistochemistry for CD20, a B-cell marker (200X).

The patient's clinical course progressed to coma due to cerebral edema and hematoma expansion. A right temporoparietal brain biopsy was obtained. Sections stained with hematoxylin-and-eosin showed small cortical vessels pathologically distended with neoplastic lymphocytes. These cells were pleomorphic, hyperchromatic, and displayed prominent nucleoli and occasional mitoses (Figure 4(a)). They did not spill out into the surrounding neuropil. Immunohistochemistry revealed these malignant cells to be diffusely positive for CD20, a B-cell marker (Figure 4(b)). The tumor cells also labeled widely for Mindbomb E3 Ubiquitin Protein Ligase 1 (MIB-1), a marker of proliferation. Based on these findings, the patient was diagnosed with IVL. The patient was urgently started on chemotherapy with rituximab, cyclophosphamide, doxorubicin, vincristine, and prednisone (R-CHOP) along with decompressive craniectomy, which led to reversal of coma and liberation from the ventilator several days later. She then received methotrexate, which halted progression of her disease and allowed her to return home with a modified Rankin Scale of 1. The patient remains in remission today, four years later.

## 3. Discussion

IVL involving the CNS is an aggressive condition with a poor prognosis because its protean clinical manifestations frequently preclude diagnosis. Laboratory and imaging tools provide clues to the etiology, but lack adequate sensitivity and specificity. Our patient exhibited an unusual presentation of multiple arterial dissections and progressive ICH. She underwent an extensive workup for progressive encephalopathy until her decline into coma necessitated a brain biopsy. The pathological proliferation of intraluminal lymphocytes in the small intracranial vessels explains our patient's symptoms and imaging findings. As arterioles, venules, and capillaries stagnate with malignant cells, intraparenchymal hemorrhage and infarction can occur. However, the formation of multiple large-artery dissections in her clinical course remains unexplained. Microvessels are a known source of intramural hematomas in the setting of arterial dissection and intraplaque hemorrhage, attributed to vasa vasorum leakage or rupture within the wall of carotid and vertebral arteries [12]. It is unclear whether IVL is capable of infiltrating these small vessels (vasa vasorum) within the arterial wall to cause rupture and dissection of the intima and adventitia of the artery.

Given the limitations of an individual reported case, we cannot rule out an alternative etiology for her dissections. It is possible that she had either underlying fibromuscular dysplasia (FMD) or another condition explaining her dissections, in addition to IVL explaining her brain findings. However, her angiographic studies showed interval development of new large-artery dissections associated with clinical and radiographic progression of her IVL. To date, there have been no reports in the literature to support IVL involving large arteries such as carotid and vertebral arteries, a disease known to involve mainly arterioles, venules, and capillaries.

This case highlights the need for a high index of suspicion for an underlying IVL in the setting of multiple intracranial and extracranial dissections and ICH, and progression of findings despite standard medical therapy. Our patient recovered from her coma and currently remains in remission four years later owing to accurate histopathological diagnosis and prompt treatment, which stands in stark contrast to the majority of patients who are diagnosed postmortem. In evaluating progressive encephalitis of unclear etiology, prompt brain biopsy proved essential in directing treatment. However, there may be reasons when brain biopsy may be delayed or inaccurate due to concerns regarding procedural risk or when there is little to no tumor mass formation, which could lead to non-diagnostic results that have the potential to critically delay the initiation of therapy. Imaging findings such as diffuse scattered foci of T2 hyperintensities and diffusion restriction, petechial and parenchymal hemorrhages, with or without arterial dissections should raise the suspicion for this elusive disease even in the absence of detectable mass lesions. Early clinical diagnostic strategies may enable treatment sooner. These strategies make the diagnosis by assimilating other important data including serum tumor markers, LDH (lactate dehydrogenase) and soluble IL2R (interleukin-2 receptor), as well as random skin and bone marrow biopsy with flow cytometry [6, 8, 9, 13, 14]. In particular, CNS involvement of lymphoma at diagnosis portends a worse prognosis and these patients are thought to have a higher rate of relapse, especially in the CNS [15]. It has been proposed that positive findings on skin biopsy may be useful in cases without proven CNS disease as a risk factor for later relapse in the CNS, though further investigation is needed to prove this [15]. There remains a need for better pathophysiologic understanding of this elusive disease.

## Figures and Tables

**Table 1:** Additional laboratory findings.

	Value	Interpretation
*Comprehensive metabolic panel*		
Sodium	142 mmol/L	
Potassium	3.6 mmol/L	
Chloride	108 mmol/L	
CO_2_	26 mmol/L	
BUN	10 mg/dL	
Creatinine	1.24 mg/dL	^∗^High
Glucose	103 mg/dL	
Calcium	9.2 mg/dL	
Protein, total	6.1 g/dL	^∗^Low
Albumin	4.1 g/dL	
Bilirubin	0.7 mg/dL	
Alkaline phosphatase	60 U/L	
AST	17 U/L	
ALT	11 U/L	
Anion gap	8 mmol/L	

*Complete blood count*		
WBC	3.73 k/*µ*l	
RBC	4.47 M/*µ*l	
HGB	13.3 g/dL	
HCT	38.9%	
MCV	86.9 fL	
MCH	29.7 pg	
MCHC	34.2 g/dL	
RDW	14.8%	^∗^High
Platelet	152 k/*µ*l	^∗^Low
MPV	6.9 fL	
Granulocyte %, #	63.4, 2.4 k/*µ*l	
Monocyte %, #	9.1, 0.3 k/*µ*l	%^∗^High
Eosinophil %, #	3.1, 0.1 k/*µ*l	
Basophil %, #	0.4, 0.0 k/*µ*l	
Lymphocyte %, #	24, 0.9 k/*µ*l	

*Paraneoplastic autoantibody evaluation (CSF)*		
Neuronal nuclear Ab, Type 1	Negative	
Neuronal nuclear Ab, Type 2	Negative	
Neuronal nuclear Ab, Type 3	Negative	
Glial Nuclear Ab	Negative	
Purkinje cell cytoplasmic Ab, Type 1	Negative	
Purkinje cell cytoplasmic Ab, Type 2	Negative	
Purkinje cell cytoplasmic Ab, Type Tr	Negative	
Amphiphysin Ab	Negative	
CRMP-5 IgG	Negative	

*Additional laboratory tests*		
LDH^a^	427 U/L	^∗^High
Ferritin^b^	1,107 ng/mL	^∗^High
TSH	1.83 mU/L	
CRP	0.1 mg/dL	
Prothrombin time	13.7 sec	
INR	1.1	
Sed rate, Westergren	2 mm/hr	

^a^LDH was analyzed 45 days after initial test results. ^b^Ferritin was analyzed 20 months after initial test results.

**Table 2:** Laboratory/imaging workup.

Differential diagnosis	Labs/imaging
Neurosarcoidosis	ACE
Cerebral sinus thrombosis	Catheter angiogram
Vasculitis	ANA, ANCA, SSA, SSB, ESR, CRP, Hepatitis panel
Reversible cerebral vasoconstriction syndrome	Catheter angiogram and high resolution vessel wall MRI
Autoimmune and paraneoplastic encephalitis	VGKC, NMDA, CASPR2, LGI1 paraneoplastic antibody panel, Cerebral FDG PET scan
CNS malignancy or metastasis	CSF cytology and flow cytometry, CT of the chest, abdomen, pelvis. PET of the brain
Infectious endocarditis with septic emboli	ESR, CRP, lactate
Encephalitis (Infectious etiologies)	TB, RPR, HIV, VZV, HSV, EBV, WNV, HTLV1/2, and others
Cerebral autosomal dominant arteriopathy with subcortical infarcts and leukoencephalopathy (CADASIL)	MRI
Drug-induced vasculopathy	Urine drug screen
Amyloid angiitis	Congo red and thioflavin on biopsy, MRI with Susceptibility-weighted imaging
Progressive multifocal leukoencephalopathy	HIV antibodies
